# Commentary: Neutral Commentary on Frontiers Article “Cobalt Chloride Upregulates Impaired HIF-1α Expression to Restore Sevoflurane Post-conditioning-Dependent Myocardial Protection in Diabetic Rats”

**DOI:** 10.3389/fphys.2017.00926

**Published:** 2017-11-21

**Authors:** Nila Ghanei, Rajesh H. Amin

**Affiliations:** Cardio-Metabolic Research Lab, Department of Drug Discovery and Development, The Harrison School of Pharmacy, Auburn University, Auburn, AL, United States

**Keywords:** Hypoxia-Inducible Factor 1, alpha subunit, myocardial infarction, oxidative phosphorylation, diabetes mellitus, Type 2, sevoflurane post-conditioning

Coronary heart diseases, including acute myocardial infarction (MI), accounts for high mortality rates in the United States. In addition, diabetes exists as a comorbidity for the majority of all myocardial infarction mortalities (Kannel and McGee, 1979; Danaei et al., 2009) due from occluded coronary circulation, myocardial energy dysregulation, and insulin resistance (Wells et al., 2013). The prognosis and mortality rates from MI depend on the infarction size, which also determines the rate of progression to heart failure (Pfeffer and Braunwald, 1990). Therefore, understanding the molecular signaling cascades that occur in the myocardium due to myocardial infarction is necessary to identify novel therapeutic targets to mitigate the cardiac damage and progression to heart failure. Previous reports have observed the pre- and post-conditioning of sevoflurane upon the heart for protection against ischemia reperfusion (IR) injury involved PI3K-AKT, ERK1/2, and GSK3β signaling pathway (Cadenas et al., 2010). However, more recent studies have focused upon the beneficial effects of Hypoxia inducible factor 1 alpha (HIF-α) cardioprotective signaling mechanism (Hwang et al., 2015; Nanayakkara et al., 2015). Recent studies utilizing an intermittent hypoxia technique in a Hypoxia Inducible Factor-1 (HIF-1α) knockout mouse implicated HIF-1α to be the critical mediator of ischemic pre and post-conditioning (Eckle et al., 2008; Zhao et al., 2010). These reports validate the significance of HIF-1α in conditioning via stabilization of HIF-1α (Zhao et al., 2010). The current manuscript titled,” Cobalt Chloride Upregulates Impaired HIF-1-alpha Expression to Restore Sevoflurane Post-conditioning-Dependent Myocardial Protection in Diabetic Rats “ by Wu et al., in the journal Frontiers Physiology, 2017 June 13;8:395, further investigates the scope of HIF-1α signaling in the cardioprotective sevoflurane signaling against IR injury in the diabetic heart (Wu et al., 2017).

HIF-1 is a master transcriptional regulator of hypoxia-inducible genes and is a heterodimer consisting of α and β subunits (Ke and Costa, 2006). In the presence of oxygen, HIF-1α protein is hydroxylated on conserved prolyl residues by members of the egg-laying-defective 9 (EglN) prolyl hydroxylases (PHD) (Bruick and McKnight, 2001; Epstein et al., 2001; Ivan et al., 2001; Jaakkola et al., 2001; Yu et al., 2001). In response to low oxygen, HIF-1α participates in many essential cellular survival processes by migrating to the nucleus where it interacts with HIF-1β and induces transcriptional activation of key target genes that regulate energy metabolism and angiogenesis (Ke and Costa, 2006). Most importantly the primary adaptive significance of HIF-1α under hypoxic stress is to maintain redox homeostasis by remodeling myocardial energy related processes including increased glucose utilization and decreased respiration (Zhao et al., 2010). However, the intrinsic cardioprotective mechanisms of pre- and post-conditioning mechanisms have been shown to be attenuated by the diabetic condition, resulting in enhanced myocardial damage in response to ischemic stress. More specifically, it has been reported that diabetes attenuates the HIF-1α mediated cardioprotection against IR injury. Work by Thangarajah et al., have shown that the advanced glycation end products associated from diabetes, prevents HIF-1α mediated regulation of vascular endothelial growth factor (VEGF) resulting in diminished neovascular collateral circulation (Thangarajah et al., 2018). They determined that methylglyoxal an advanced glycation intermediate interacts with the HIF-1α cofactor CBP/P300 thus preventing HIF-1α from interacting with HIF-1β resulting in attenuating the transcriptional regulation of VEGF.

The current study by Wu et al., investigates the role sevoflurane as a post-conditioning mechanism against IR injury in a diabetic rat model (Wu et al., 2017). The cardioprotective signaling mechanism offered by the investigators determines that sevoflurane promotes stabilization of HIF-1α resulting in mitigating IR injury. However, they observed that in a type-2 diabetic rat model, this protective mechanism was attenuated. To further evaluate a potential mitigating factor associated with diabetes, the investigators negated the involvement of hyperglycemia upon altering HIF-1α activity by reducing circulating blood glucose levels with the administration of exogenous insulin. However, ameliorating circulating blood glucose levels had no impact on improving sevoflurane mediated HIF-1α stabilization. PHDs are centrally involved in modifying (hydroxylation) HIF-1α which results in HIF-1α proteasomal degradation. The investigators utilized cobalt chloride to inhibit the PHDs and thus promoted sevoflurane's cardio-protective mechanism. Cobalt chloride has been observed to induce the transcriptionally activity of HIF-1α by the production of reactive oxygen species (ROS) and the operation of the phosphatidylinositol-3 kinase (PI-3K) and MAPK pathways (Triantafyllou et al., 2006). However, alternative studies have observed oxidative mediated tissue damage with chronic cobalt chloride administration (Lippi et al., 2005).

IR injury induces large amounts of reactive oxygen species (ROS). These ROS are central mediators for damage to the myocardium which results in injury to the mitochondria and decreased levels of oxidative phosphorylation. However, the source of this ROS remains elusive and further studies demonstrating how sevoflurane mitigates ROS formation, will help in defining the cardioprotective mechanism of sevoflurane against IR injury. HIF-1α is considered critical for metabolic adaptation to hypoxia through increased conversion of glucose to pyruvate and subsequently to lactate (Kim et al., 2006). HIF-1α has also been observed to suppress metabolism through the tricarboxylic acid cycle (TCA) by directly trans-activating the gene encoding pyruvate dehydrogenase kinase 1 (PDK1) (Kim et al., 2006). PDK1 inactivates the TCA cycle enzyme, pyruvate dehydrogenase (PDH), which converts pyruvate to acetyl-CoA. The increased PDK1 expression induces an increase in ATP levels, attenuates hypoxic ROS generation, and rescues cells from hypoxia-induced apoptosis (Kim et al., 2006). The hypoxia-induced metabolic switch shunts glucose metabolites from the mitochondria to glycolysis to maintain ATP production and to prevent ROS production. Conversely, work by Hwang et al report that HIF-1α regulates mitochondrial oxygen consumption and upregulates nuclear encoded electron transport genes (Hwang et al., 2015). The current manuscript further supports the concept that HIF-1α enhances the mitochondrial electron transport chain. Remarkably, cobalt chloride enhances the post-conditioning of sevoflurane- HIF-1α activity upon the mitochondrial state III including enzymes intricately involved in oxidative phosphorylation such as NADH Oxidase, Cytochrome-C Oxidase, and Succinate Oxidase, while reducing mitochondrial ROS formation. These findings suggest an alternative cardioprotective signaling mechanism offered by sevoflurane- HIF-1α signaling against IR injury. This may include elevation of endothelial nitric oxide synthase (eNOS) and VEGF expression which results in reducing ROS levels in the infarcted myocardium. Alternatively, work by Nanayakkara et al. have observed that HIF-1α protects against IR-induced mitochondrial ROS formation, cellular energy dysregulation and prevents cellular demise by regulating ferrous iron accumulation and the mitochondrial reactive oxygen species that form via the Fenton reaction (Nanayakkara et al., 2015). Further investigation involving pre and post conditioning of sevoflurane upon ameliorating mitochondrial energy dysregulation and ROS formation for cardioprotection against IR injury in diabetic patients will improve the applicability of sevoflurane as a therapeutic agent for myocardial infarction.

## Author contributions

All authors listed have made a substantial, direct and intellectual contribution to the work, and approved it for publication.

### Conflict of interest statement

The authors declare that the research was conducted in the absence of any commercial or financial relationships that could be construed as a potential conflict of interest. The reviewer JQ declared a past collaboration with one of the authors RA to the handling Editor
